# Supplementary material for: An open-source framework for large-scale, flexible evaluation of biomedical text mining systems
Source: J Biomed Discov Collab. 2008 Jan 29;3:1. doi: 10.1186/1747-5333-3-1 (PMC2276192; doi:10.1186/1747-5333-3-1)
Supplement: Additional file 1 — Evaluation platform source code. The additional file contains the software evaluation framework discussed in this paper. The most current release can be downloaded from SourceForge.net [1]. See the accompanying README file for instructions on installation and use. Also included in the distribution is a collection of UIMA wrappers for some commonly used BioNLP tools and annotated corpora. [file 1747-5333-3-1-S1.zip › bionlp_uima_repository-1.0.1/docs/api/src-html/edu/uchsc/ccp/util/nlp/annotation/TextAnnotation.html]

```
001    /*
002     *
003     * The Original Code is UCHSC Utilities.
004     *
005     * The Initial Developer of the Original Code is University of Colorado.  
006     * Copyright (C) 2005.  All Rights Reserved.
007     *
008     * UCHSC Utilities was developed by the Center for Computational Pharmacology
009     * (http://compbio.uchsc.edu) at the University of Colorado Health 
010     *  Sciences Center School of Medicine with support from the National 
011     *  Library of Medicine.  
012     *
013     *
014     * Contributor(s):
015     *   William A. Baumgartner <william.baumgartner@uchsc.edu> (Original Author)
016     *   
017     * Created: September 2005
018     * Dependencies:
019     * Changes:
020     *      11/28/05 - wab - Extended class mention storage to incorporate complext slot values 
021     *                       i.e. slot values that are themselves class mentions) 
022     *                       classMentionMap now stores ClassMention objects instead of Strings
023     *      01/05/06 - wab - Enabled association with multiple spans: getSpans(), setSpans()
024     *                     - Enabled association with multiple annotation sets: getAnnotationSets(), setAnnotationSets()
025     *                     - Deprecated getBeginIndex(), setBeginIndex(), getEndIndex(), setEndIndex(), getAnnotationSetID(), setAnnotationSetID()
026     *      01/11/06 - wab - Changed DocumentID to a String to make it more flexible
027     *      04/30/06 - wab - added getSingleLineRepresentation()
028     *      06/07/06 - wab - span list is now stored in sorted order
029     *                     - updated equals()
030     *                     - added overlaps()
031     *      03/28/07 - wab - major overhaul of the TextAnnotation class - removed many deprecated methods, changed a bunch of stuff 
032     *                       to comply with the newly generated annotation comparison machinery
033     */
034    
035    package edu.uchsc.ccp.util.nlp.annotation;
036    
037    import java.io.PrintStream;
038    import java.util.ArrayList;
039    import java.util.Collections;
040    import java.util.HashSet;
041    import java.util.List;
042    import java.util.Set;
043    
044    import edu.uchsc.ccp.util.nlp.annotation.comparison.AnnotationComparator;
045    import edu.uchsc.ccp.util.nlp.mention.ClassMention;
046    
047    /**
048     * This class is a generalized representation of a text annotation.
049     * 
050     * @author William A. Baumgartner, Jr.
051     */
052    
053    public class TextAnnotation implements Comparable {
054        // private final boolean DEBUG = false;
055    
056        private List<Span> spanList;
057    
058        private Annotator annotator;
059    
060        private Set<AnnotationSet> annotationSets;
061    
062        private String coveredText; // text covered by the span
063    
064        private int annotationID; // annotation ID
065    
066        private String documentID; // document ID
067    
068        private int documentCollectionID; // document collection ID, see
069    
070        private int documentSectionID; // document section ID, see Annotation
071    
072        private ClassMention classMention;
073    
074      /**
075       * 
076       * @param beginIndex
077       * @param endIndex
078       * @param coveredText
079       * @param annotator
080       * @param annotationSet
081       * @param annotationID
082       * @param documentCollectionID
083       * @param documentID
084       * @param documentSectionID
085       * @param classMention
086       */
087        public TextAnnotation(int beginIndex, int endIndex, String coveredText, Annotator annotator, AnnotationSet annotationSet,
088                int annotationID, int documentCollectionID, String documentID, int documentSectionID, ClassMention classMention) {
089    
090            Span span;
091            try {
092                span = new Span(beginIndex, endIndex);
093                this.spanList = new ArrayList<Span>();
094                spanList.add(span);
095            } catch (InvalidSpanException e) {
096                e.printStackTrace();
097            }
098    
099            this.coveredText = coveredText;
100            this.annotator = annotator;
101    
102            this.annotationSets = new HashSet<AnnotationSet>();
103            annotationSets.add(annotationSet);
104    
105            this.annotationID = annotationID;
106            this.documentCollectionID = documentCollectionID;
107            this.documentID = documentID;
108            this.documentSectionID = documentSectionID;
109            this.setClassMention(classMention);
110        }
111    
112        /**
113         * Default Constructor - creates default TextAnnotation - I would like to phase this out, therefore the Deprecated flag.
114         * 
115         * Default values: beginIndex = -1 <br>
116         * endIndex = -1 <br>
117         * coveredText = empty<br>
118         * String annotatorID = -1 <br>
119         * annotationSetID = -1 <br>
120         * annotationID = -1 <br>
121         * documentCollectionID = -1 <br>
122         * documentID = -1 <br>
123         * documentSectionID = -1 <br>
124         * classMentionMap = empty HashMap
125         */
126        @Deprecated
127        public TextAnnotation() {
128            Span span;
129            this.spanList = new ArrayList<Span>();
130            try {
131                span = new Span(-1, -1);
132                spanList.add(span);
133            } catch (InvalidSpanException e) {
134                e.printStackTrace();
135            }
136            this.coveredText = "";
137            this.annotator = new Annotator(new Integer(-1), "", "", "");
138            this.annotationSets = new HashSet<AnnotationSet>();
139            this.annotationID = -1;
140            this.documentCollectionID = -1;
141            this.documentID = "-1";
142            this.documentSectionID = -1;
143            this.classMention = null;
144        }
145    
146        /*************************************************************************************************************************************************
147         * GETTERS AND SETTERS
148         ************************************************************************************************************************************************/
149    
150        /**
151         * This method returns the corresponding annotator ID
152         * 
153         * @return annotator ID
154         */
155        public int getAnnotatorID() {
156            return annotator.getAnnotatorID().intValue();
157        }
158    
159        /**
160         * This method sets the annotator ID
161         * 
162         * @param annotatorID
163         */
164        @Deprecated
165        public void setAnnotatorID(int annotatorID) {
166            this.annotator.setAnnotatorID(new Integer(annotatorID));
167        }
168    
169        /**
170         * Set the annotator
171         * 
172         * @return
173         */
174        public Annotator getAnnotator() {
175            return annotator;
176        }
177    
178        /**
179         * Get the annotator
180         * 
181         * @param annotator
182         */
183        public void setAnnotator(Annotator annotator) {
184            this.annotator = annotator;
185        }
186    
187        /**
188         * Get the annotation sets
189         * 
190         * @return
191         */
192        public Set<AnnotationSet> getAnnotationSets() {
193            return annotationSets;
194        }
195    
196        /**
197         * Add an annotation set
198         * 
199         * @param annotationSet
200         */
201        public void addAnnotationSet(AnnotationSet annotationSet) {
202            annotationSets.add(annotationSet);
203        }
204    
205        /**
206         * Return a set containing the AnnotationSet IDs for which this TextAnnotation is a member.
207         * 
208         * @return
209         */
210        public Set<Integer> getAnnotationSetIDs() {
211            Set<Integer> annotationSetIDs = new HashSet<Integer>();
212    
213            for (AnnotationSet annotationSet : annotationSets) {
214                annotationSetIDs.add(annotationSet.getAnnotationSetID());
215            }
216    
217            return annotationSetIDs;
218        }
219    
220        /**
221         * Set the AnnotationSet membership for this TextAnnotation
222         * 
223         * @param annotationSets
224         */
225        public void setAnnotationSets(Set<AnnotationSet> annotationSets) {
226            this.annotationSets = annotationSets;
227        }
228    
229        /**
230         * Return true if this TextAnnotation is a member of the inputted annotation set, false otherwise.
231         * 
232         * @param annotationSetID
233         * @return
234         */
235        public boolean isMemberOfAnnotationSet(int annotationSetID) {
236            return getAnnotationSetIDs().contains(annotationSetID);
237        }
238    
239        /**
240         * Get the annotation ID
241         * 
242         * @return
243         */
244        public int getAnnotationID() {
245            return annotationID;
246        }
247    
248        /**
249         * Set the annotation ID
250         * 
251         * @param annotationID
252         */
253        public void setAnnotationID(int annotationID) {
254            this.annotationID = annotationID;
255        }
256    
257        /**
258         * Get the document collection ID
259         * 
260         * @return
261         */
262        public int getDocumentCollectionID() {
263            return documentCollectionID;
264        }
265    
266        /**
267         * Set the document collection ID
268         * 
269         * @param documentCollectionID
270         */
271        public void setDocumentCollectionID(int documentCollectionID) {
272            this.documentCollectionID = documentCollectionID;
273        }
274    
275        /**
276         * Get the document ID
277         * 
278         * @return
279         */
280        public String getDocumentID() {
281            return documentID;
282        }
283    
284        /**
285         * Set the document ID
286         * 
287         * @param documentID
288         */
289        public void setDocumentID(String documentID) {
290            this.documentID = documentID;
291        }
292    
293        /**
294         * Get the document section ID
295         * 
296         * @return
297         */
298        public int getDocumentSectionID() {
299            return documentSectionID;
300        }
301    
302        /**
303         * Set the document section ID
304         * 
305         * @param documentSectionID
306         */
307        public void setDocumentSectionID(int documentSectionID) {
308            this.documentSectionID = documentSectionID;
309        }
310    
311        /**
312         * Return the text that is "covered" by this annotation
313         */
314        public String getCoveredText() {
315            return coveredText;
316        }
317    
318        /**
319         * Set the text that is "covered" by this annotation
320         * 
321         * @param coveredText
322         */
323        public void setCoveredText(String coveredText) {
324            this.coveredText = coveredText;
325        }
326    
327        /**
328         * Get the ordered list of spans for this TextAnnotation
329         * 
330         * @return
331         */
332        public List<Span> getSpans() {
333            return this.spanList;
334        }
335    
336        /**
337         * Set the span list equal to the inputted span list
338         * 
339         * @param spans
340         */
341        public void setSpans(List<Span> spans) {
342            spanList = new ArrayList<Span>();
343            for (Span span : spans) {
344                addSpan(span);
345            }
346        }
347    
348        /**
349         * Set the annotation span to be equal to the single input span.
350         * 
351         * @param span
352         */
353        public void setSpan(Span span) {
354            this.spanList = new ArrayList<Span>();
355            spanList.add(span);
356        }
357    
358        /**
359         * Add a span to this TextAnnotation. The span will be placed in the proper position in the ordered span list.
360         * 
361         * @param span
362         */
363        public void addSpan(Span span) {
364            boolean addedSpan = false;
365            int spanListSize = spanList.size();
366    
367            if (spanListSize == 0) {
368                /* then just add the span */
369                spanList.add(span);
370            } else {
371                /* add the span, in sorted order */
372                for (int i = 0; i < spanListSize; i++) {
373                    Span sortedSpan = spanList.get(i);
374                    if (span.getSpanStart() < sortedSpan.getSpanStart()) {
375                        /* then we need to add this span in front of the one in the list */
376                        spanList.add(i, span);
377                        addedSpan = true;
378                        break;
379                    } else if (span.getSpanStart() == sortedSpan.getSpanStart()) {
380                        /* if span starts are equal, check span ends. If the new span ends before the sorted span, then add it in front */
381                        if (span.getSpanEnd() < sortedSpan.getSpanEnd()) {
382                            spanList.add(i, span);
383                            addedSpan = true;
384                            break;
385                        }
386                    }
387                    /* span has not been added, so add at end of list */
388                    if (!addedSpan) {
389                        spanList.add(spanList.size(), span);
390                    }
391                }
392            }
393            /* if there are any [-1..-1] spans then remove them, these spans are added by the default constructor */
394            try {
395                Span defaultSpan = new Span(-1, -1);
396                spanList.remove(defaultSpan);
397            } catch (InvalidSpanException e) {
398                e.printStackTrace();
399            }
400    
401        }
402    
403        // @Deprecated
404        // public int getAnnotationSpanStart() {
405        // Span span = this.spanList.get(0);
406        // return span.getSpanStart();
407        // }
408        //
409        // @Deprecated
410        // public void setAnnotationSpanStart(int startIndex) throws InvalidSpanException {
411        // this.spanList.get(0).setSpanStart(startIndex);
412        // }
413        //
414        // @Deprecated
415        // public int getAnnotationSpanEnd() {
416        // Span span = this.spanList.get(0);
417        // return span.getSpanEnd();
418        // }
419        //
420        // @Deprecated
421        // public void setAnnotationSpanEnd(int endIndex) throws InvalidSpanException {
422        // this.spanList.get(0).setSpanEnd(endIndex);
423        // }
424    
425        /**
426         * Return the start index of the first span (spans are stored in order)
427         * 
428         * @return
429         */
430        public int getAnnotationSpanStart() {
431            return this.spanList.get(0).getSpanStart();
432        }
433    
434        /**
435         * Sets the start index for the first annotation span
436         * 
437         * @param spanStart
438         */
439        public void setAnnotationSpanStart(int spanStart) {
440            int currentSpanStart = this.getAnnotationSpanStart();
441            try {
442                this.spanList.get(0).setSpanStart(spanStart);
443            } catch (InvalidSpanException ise) {
444                error("Invalid span. ["
445                        + spanStart
446                        + ".."
447                        + this.spanList.get(0).getSpanEnd()
448                        + "] --- this error is often caused by setting the span start prior to setting the span end, please check your code to see if this is the case. --- Annotation span reverted to last known safe state.");
449                ise.printStackTrace();
450                /* return the span to it's previous state */
451                try {
452                    this.spanList.get(0).setSpanStart(currentSpanStart);
453                } catch (InvalidSpanException ise2) {
454                    ise2.printStackTrace();
455                    /* This exception should never be thrown as we are setting the start index back to the previous state (which should have been safe) */
456                }
457            }
458        }
459    
460        /**
461         * Return the end index of the last span (spans are stored in order)
462         */
463        public int getAnnotationSpanEnd() {
464            return spanList.get(spanList.size() - 1).getSpanEnd();
465        }
466    
467        /**
468         * Set the end index for the last annotation span
469         * 
470         * @param spanEnd
471         */
472        public void setAnnotationSpanEnd(int spanEnd) {
473            int currentSpanEnd = this.getAnnotationSpanEnd();
474            try {
475                this.spanList.get(spanList.size() - 1).setSpanEnd(spanEnd);
476            } catch (InvalidSpanException ise) {
477                error("Invalid span. [" + this.spanList.get(spanList.size() - 1).getSpanStart() + ".." + spanEnd
478                        + "] --- Annotation span reverted to last known safe state.");
479                ise.printStackTrace();
480                /* return the span to it's previous state */
481                try {
482                    this.spanList.get(spanList.size() - 1).setSpanEnd(currentSpanEnd);
483                } catch (InvalidSpanException ise2) {
484                    ise2.printStackTrace();
485                    /* This exception should never be thrown as we are setting the start index back to the previous state (which should have been safe) */
486                }
487            }
488        }
489    
490        public ClassMention getClassMention() {
491            return classMention;
492        }
493    
494        /**
495         * When setting the classmention, the a reference to the text annotation is automatically created inside the classMention object.
496         * 
497         * @param classMention
498         */
499        public void setClassMention(ClassMention classMention) {
500            this.classMention = classMention;
501            // always reference the text annotation that the class mention belongs to
502            classMention.addTextAnnotation(this);
503        }
504    
505        /**
506         * Return a String that can be used as a hashkey for this TextAnnotation - I wonder if this should be deprecated?!
507         * 
508         * @return
509         */
510        @Deprecated
511        public String getHashKey() {
512            // key = annotatorID mentionName docID docSectionID spanBegin spanEnd ...
513            String mentionName = "";
514            try {
515                mentionName = this.getClassMention().getMentionName();
516            } catch (NullPointerException npe) {
517    
518            }
519            String key = Integer.toString(this.getAnnotatorID()) + " " + mentionName + " " + this.getDocumentID() + " "
520                    + this.getDocumentSectionID() + " ";
521            ArrayList<Span> spans = (ArrayList<Span>) this.getSpans();
522            for (edu.uchsc.ccp.util.nlp.annotation.Span span : spans) {
523                key += (Integer.toString(span.getSpanStart()) + " ");
524                key += (Integer.toString(span.getSpanEnd()) + " ");
525            }
526    
527            return key;
528        }
529    
530        /**
531         * Output a representation of this TextAnnotation to the input PrintStream
532         * 
533         * @param ps
534         */
535        public void printAnnotation(PrintStream ps) {
536            String spansStr = "";
537            for (Span span : spanList) {
538                spansStr += (span.getSpanStart() + " - " + span.getSpanEnd() + "  ");
539            }
540            ps.println("======================= Annotation: " + annotationID + " =======================");
541            ps.print("Annotator: ");
542            if (annotator != null) {
543                this.annotator.printStorageLine(ps);
544            } else {
545                ps.println("NULL");
546            }
547            ps.print("--- AnnotationSets: ");
548            for (AnnotationSet annotationSet : annotationSets) {
549                if (annotationSet != null) {
550                    annotationSet.printStorageLine(ps);
551                } else {
552                    System.err.println("WARNING -- TextAnnotation: Null Annotation Set found inside TextAnnotation...");
553                }
554            }
555            ps.println("--- Span: " + spansStr);
556            ps.println("--- DocCollection: " + documentCollectionID + "  DocID: " + documentID + "  DocumentSection: " + documentSectionID);
557            ps.println("--- Covered Text: " + coveredText);
558    
559            ClassMention.printMention(this.classMention, ps, 0);
560            ps.println("=================================================================================");
561        }
562    
563        /**
564         * return a String which represents this annotation, including all class mentions and any associated annotations
565         * 
566         * NOTE: ANY CHANGES HERE MUST BE MIRRORED IN Knowtator_Util.getSingleLineRepresentationOfAnnotation()
567         * 
568         * and probably UIMA_Util
569         * 
570         * @return
571         */
572        public String getSingleLineRepresentation(boolean showDocID, boolean deep) {
573            String repStr;
574    
575            if (showDocID) {
576                repStr = documentID + "|";
577            } else {
578                repStr = "";
579            }
580    
581            String spansStr = "";
582            for (Span span : spanList) {
583                spansStr += ("[" + span.getSpanStart() + ".." + span.getSpanEnd() + "]");
584            }
585            spansStr = spansStr.substring(0, spansStr.length());
586    
587            String annotationSetStr = "";
588            List<Integer> aSetIDs = Collections.list(Collections.enumeration(this.getAnnotationSetIDs()));
589            Collections.sort(aSetIDs);
590            for (Integer aSetID : aSetIDs) {
591                annotationSetStr += (aSetID + ",");
592            }
593    
594            if (deep) {
595                repStr += (spansStr + "|\"" + coveredText + "\"" + "|" + annotator.getLastName() + "|" + annotationSetStr + "{"
596                        + classMention.getSingleLineRepresentation() + "}");
597            } else {
598                repStr += (spansStr + "|\"" + coveredText + "\"" + "|" + annotator.getLastName() + "|" + annotationSetStr);
599            }
600    
601            return repStr;
602        }
603    
604        /**
605         * Same a single line representation, however no span, covered text, or annotation meta info, is shown.. just the class mention
606         * 
607         * @param showDocID
608         * @param deep
609         * @return
610         */
611        public String getDocumentLevelSingleLineRepresentation(boolean deep) {
612            String repStr;
613    
614            String annotationSetStr = "";
615            List<Integer> aSetIDs = Collections.list(Collections.enumeration(this.getAnnotationSetIDs()));
616            Collections.sort(aSetIDs);
617            for (Integer aSetID : aSetIDs) {
618                annotationSetStr += (aSetID + ",");
619            }
620            
621            repStr = documentID + "|" + annotationSetStr + "|";
622    
623            if (deep) {
624                repStr += ("{" + classMention.getDocumentLevelSingleLineRepresentation() + "}");
625            }
626    
627            return repStr;
628        }
629    
630        /**
631         * simple output on one line.. will not print complex slot mentions
632         * 
633         * @param ps
634         */
635        public void printAnnotationOnOneLine(PrintStream ps) {
636            // String annotationStr = "";
637            // String spansStr = "";
638            // for (Span span : spanList) {
639            // spansStr += ("[" + span.getSpanStart() + ".." + span.getSpanEnd() + "]:");
640            // }
641            // spansStr = spansStr.substring(0,spansStr.length());
642            //        
643            // annotationStr = (documentID + "|" + spansStr + "|" + classMention.getMentionName() + "|" + coveredText + "|" +
644            // annotator.getFirstName());
645            // ps.println(annotationStr);
646    
647            ps.println(getSingleLineRepresentation(true, true));
648        }
649    
650        public int compareTo(Object textAnnotationToCompare) {
651            if (!(textAnnotationToCompare instanceof TextAnnotation)) {
652                throw new ClassCastException("A TextAnnotation object expected.");
653            } else {
654                AnnotationComparator ac = new AnnotationComparator();
655                return ac.compare(this, (TextAnnotation) textAnnotationToCompare);
656            }
657        }
658    
659        /**
660         * The default equals() method requires exact span match, as well as identical class mention match to return true.
661         */
662        @Override
663        public boolean equals(Object textAnnotationToEquate) {
664            if (!(textAnnotationToEquate instanceof TextAnnotation)) {
665                throw new ClassCastException("A TextAnnotation object expected.");
666            } else {
667                TextAnnotation ta = (TextAnnotation) textAnnotationToEquate;
668    
669                if (this.compareTo(ta) == 0) {
670                    return true;
671                } else {
672                    return false;
673                }
674            }
675        }
676    
677        /**
678         * Returns the total span of this annotation (i.e. from the start of the first span to the end of the last span)
679         */
680        public Span getAggregateSpan() {
681            try {
682                Span totalSpan = new Span(this.getAnnotationSpanStart(), this.getAnnotationSpanEnd());
683                return totalSpan;
684            } catch (InvalidSpanException e) {
685                /*
686                 * This should never throw an exception since the spans will have been set previously. If it does throw an exception here, then the
687                 * sorting mechanism for the spans is most likely to blame.
688                 */
689                e.printStackTrace();
690                return null;
691            }
692        }
693    
694        /**
695         * returns true if the total span of this TextAnnotation overlaps with the total span of the input TextAnnotation
696         * 
697         * @return
698         */
699        public boolean overlaps(TextAnnotation ta) {
700            return this.getAggregateSpan().overlaps(ta.getAggregateSpan());
701        }
702    
703        @Override
704        public int hashCode() {
705            return getSingleLineRepresentation(true, true).hashCode();
706        }
707    
708        /**
709         * Return the length of this TextAnnotation. This method returns the length of the aggregate span.
710         * 
711         * @return
712         */
713        public int length() {
714            return this.getAggregateSpan().length();
715        }
716    
717        private void error(String message) {
718            System.err.println("ERROR -- TextAnnotation: " + message);
719        }
720    
721        // /**
722        // * This method constructs a new TextAnnotation. This method should be deprecated in the future as the classMentionMap now stores ClassMention
723        // * objects instead of Strings.
724        // *
725        // * @param beginIndex
726        // * span beginning index
727        // * @param endIndex
728        // * span ending index
729        // * @param coveredText
730        // * text covered by the span
731        // * @param annotatorID
732        // * annotator ID, see Annotation database for reference list
733        // * @param annotationSetID
734        // * annotation set ID, see Annotation database for reference list
735        // * @param annotationID
736        // * annotation ID, see Annotation database for reference list
737        // * @param documentCollectionID
738        // * document collection ID, see Annotation database for reference list
739        // * @param documentID
740        // * document ID
741        // * @param documentSectionID
742        // * document section ID, see Annotation database for reference list
743        // * @param classMentionTypes
744        // * list of class mentions
745        // * @param classMentionSlotValues
746        // * list of slot names accompanying class mention types
747        // */
748        // @SuppressWarnings("unchecked")
749        // @Deprecated
750        // public TextAnnotation(int beginIndex, int endIndex, String coveredText, int annotatorID, int annotationSetID,
751        // String[] classMentionTypes, int annotationID, int documentCollectionID, String documentID, int documentSectionID,
752        // String[] classMentionSlotValues) {
753        //
754        // // annotationType = TextAnnotationTypes.SEMANTIC;
755        // Span span;
756        // try {
757        // span = new Span(beginIndex, endIndex);
758        // this.spanList = new ArrayList<Span>();
759        // spanList.add(span);
760        // } catch (InvalidSpanException e) {
761        // e.printStackTrace();
762        // }
763        //
764        // // this.beginIndex = beginIndex;
765        // // this.endIndex = endIndex;
766        // this.coveredText = coveredText;
767        // // this.annotatorID = annotatorID;
768        // Annotator annotator = new Annotator(new Integer(annotatorID), "", "", "");
769        // this.annotator = annotator;
770        // // this.annotationSetID = annotationSetID;
771        // annotationSets = new ArrayList<AnnotationSet>();
772        // annotationSets.add(new AnnotationSet(new Integer(annotationSetID), "", ""));
773        //
774        // this.annotationID = annotationID;
775        // this.documentCollectionID = documentCollectionID;
776        // this.documentID = documentID;
777        // this.documentSectionID = documentSectionID;
778        // this.classMentionMap = new HashMap();
779        // for (int i = 0; i < classMentionTypes.length; i++) {
780        // String key = classMentionTypes[i];
781        // String value = classMentionSlotValues[i];
782        // classMentionMap.put(key, value);
783        // }
784        // }
785    
786        // /**
787        // * This method returns a list of class mention types
788        // *
789        // * @return a list of the class mention types associated with the TextAnnotation
790        // */
791        // @Deprecated
792        // @SuppressWarnings("unchecked")
793        // public String[] getClassMentionTypes() {
794        // ArrayList cmTypes = new ArrayList(classMentionMap.keySet());
795        // return (String[]) cmTypes.toArray(new String[0]);
796        // }
797        //
798        // /**
799        // * This method returns a list of slot values corresponding the the class mention types
800        // *
801        // * @return a list of the slot values corresponding to the class mention types associated with the TextAnnotation
802        // */
803        // @Deprecated
804        // @SuppressWarnings("unchecked")
805        // public String[] getClassMentionSlotValues() {
806        // ArrayList cmSlotValues = new ArrayList(classMentionMap.values());
807        // return (String[]) cmSlotValues.toArray(new String[0]);
808        // }
809        //
810        // @Deprecated
811        // @SuppressWarnings("unchecked")
812        // public Collection<ClassMention> getClassMentions() {
813        // ArrayList<ClassMention> classMentions = new ArrayList<ClassMention>(classMentionMap.values());
814        // return classMentions;
815        // }
816        //
817        // /**
818        // * This method returns a particular slot value for an inputted class mention type
819        // *
820        // * @param cmType
821        // * the name of the class mention of interest
822        // * @return the corresponding slot value for the inputted class mention type name
823        // */
824        // @Deprecated
825        // public String getClassMentionSlotValueForType(String cmType) {
826        // if (classMentionMap.containsKey(cmType)) {
827        // return (String) classMentionMap.get(cmType);
828        // } else {
829        // return null;
830        // }
831        // }
832        //
833        // @Deprecated
834        // public ClassMention getClassMentionForType(String cmType) {
835        // if (classMentionMap.containsKey(cmType)) {
836        // return (ClassMention) classMentionMap.get(cmType);
837        // } else {
838        // return null;
839        // }
840        // }
841        //
842        // /**
843        // * This method adds a class mention type/slot value pair to the TextAnnotation
844        // *
845        // * @param type
846        // * class mention type to add
847        // * @param slotValue
848        // * corresponding slot value to add
849        // */
850        // @SuppressWarnings("unchecked")
851        // @Deprecated
852        // public void addClassMention(String type, String slotValue) {
853        // if (DEBUG) {
854        // System.err.println("Adding class mention to text annotation: type=" + type + " slot=" + slotValue + ";");
855        // }
856        // classMentionMap.put(type, slotValue);
857        // }
858        //
859        // @Deprecated
860        // public void addClassMention(String type, ClassMention cm) {
861        // if (DEBUG) {
862        // System.err.println("Adding ClassMention to text annotation: type=" + type + ";");
863        // }
864        // classMentionMap.put(type, cm);
865        // }
866        //
867        // /**
868        // * This method sets the slot value for a given class mention type
869        // *
870        // * @param type
871        // * class mention type to set
872        // * @param newValue
873        // * new slot value for the given class mention type
874        // */
875        // @SuppressWarnings("unchecked")
876        // @Deprecated
877        // public void setClassMentionSlotValueForType(String type, String newValue) {
878        // if (classMentionMap.containsKey(type)) {
879        // classMentionMap.remove(type);
880        // classMentionMap.put(type, newValue);
881        // }
882        // }
883    
884        // /**
885        // * Used before TextAnnotation could be associated with multiple Spans. This returns the start of the first span in the spanList.
886        // *
887        // * @deprecated
888        // * @return
889        // */
890        // public int getBeginIndex() {
891        // Span span = this.spanList.get(0);
892        // return span.getSpanStart();
893        // // return beginIndex;
894        // }
895        //
896        // /**
897        // * Used before TextAnnotation could be associated with multiple Spans. This method sets the start of the first span in the spanList.
898        // *
899        // * @deprecated
900        // * @param beginIndex
901        // */
902        // public void setBeginIndex(int beginIndex) throws InvalidSpanException {
903        // this.spanList.get(0).setSpanStart(beginIndex);
904        // // this.beginIndex = beginIndex;
905        // }
906        //
907        // /**
908        // * Used before TextAnnotation could be associated with multiple Spans. This returns the end of the first span in the spanList.
909        // *
910        // * @deprecated
911        // * @return
912        // */
913        // public int getEndIndex() {
914        // Span span = this.spanList.get(0);
915        // return span.getSpanEnd();
916        // // return endIndex;
917        // }
918        //
919        // /**
920        // * Used before TextAnnotation could be associated with multiple Spans. This method sets the end of the first span in the spanList.
921        // *
922        // * @deprecated
923        // * @param endIndex
924        // */
925        // public void setEndIndex(int endIndex) throws InvalidSpanException {
926        // this.spanList.get(0).setSpanStart(endIndex);
927        // // this.endIndex = endIndex;
928        // }
929    
930        // /**
931        // * Used before TextAnnotation could be associated with multiple Annotation Sets. This method returns the first Annotation Set ID in the
932        // * annotationSetList.
933        // *
934        // * @deprecated
935        // * @return
936        // */
937        // public int getAnnotationSetID() {
938        // Integer setID = annotationSets.get(0).getAnnotationSetID();
939        // return setID.intValue();
940        // // return annotationSetID;
941        // }
942        //
943        // /**
944        // * Used before TextAnnotation could be associated with multiple Annotation Sets. This method sets the first Annotation Set ID in the
945        // * annotationSetList.
946        // *
947        // * @deprecated
948        // * @param annotationSetID
949        // */
950        // public void setAnnotationSetID(int annotationSetID) {
951        // annotationSets.set(0, new AnnotationSet(new Integer(annotationSetID), "", ""));
952        // // this.annotationSetID = annotationSetID;
953        // }
954    
955        // @Override
956        // public boolean equals(Object textAnnotationToEquate) {
957        // if (!(textAnnotationToEquate instanceof TextAnnotation)) {
958        // throw new ClassCastException("A TextAnnotation object expected.");
959        // } else {
960        // TextAnnotation ta = (TextAnnotation) textAnnotationToEquate;
961        //
962        // boolean metaDataMatches = metaDataEquals(ta);
963        //
964        // // /* document IDs must be equal */
965        // // boolean equalDocumentID = this.documentID.equals(ta.getDocumentID());
966        // //
967        // // /* document collection ids must be equal */
968        // // boolean equalDocumentCollectionID = this.documentCollectionID == ta.getDocumentCollectionID();
969        // //
970        // // /* spans must be equal -- all spans */
971        // // boolean equalSpans = true;
972        // // List<Span> spans = ta.getSpans();
973        // // if (spanList.size() != spans.size()) {
974        // // equalSpans = false;
975        // // } else {
976        // // for (int i = 0; i < spanList.size(); i++) {
977        // // if (!spanList.get(i).equals(spans.get(i))) {
978        // // equalSpans = false;
979        // // break;
980        // // }
981        // // }
982        // // }
983        //
984        // /*
985        // * class mention must also be equal
986        // */
987        //
988        // boolean classMentionsMatch = this.getClassMention().equals(ta.getClassMention(), false);
989        //
990        // return metaDataMatches & classMentionsMatch;
991        // }
992        // }
993    
994        // /**
995        // * Check for equality among two TextAnnotation objects. The compareClassMentions flag is useful for recursively comparing a
996        // * TextAnnotation/ClassMention structure. It is used to avoid an infinite loop where the TextAnnotation is compared, then the respective
997        // * ClassMentions are compared, and then the TextAnnotations are compared again.
998        // *
999        // * @param ta
1000        // * @param compareClassMentions
1001        // * @return
1002        // */
1003        // public boolean equals(TextAnnotation ta, boolean compareClassMentions) {
1004        // if (compareClassMentions) {
1005        // return this.equals(ta);
1006        // } else {
1007        // boolean metaDataMatches = metaDataEquals(ta);
1008        // return metaDataMatches;
1009        // }
1010        // }
1011    
1012        // /**
1013        // * Compare the meta data of two annotations (document ID, document collection ID, and span(s))
1014        // *
1015        // * @param ta
1016        // * @return
1017        // */
1018        // public boolean metaDataEquals(TextAnnotation ta) {
1019        // /* document IDs must be equal */
1020        // boolean equalDocumentID = this.documentID.equals(ta.getDocumentID());
1021        //
1022        // /* document collection ids must be equal */
1023        // boolean equalDocumentCollectionID = this.documentCollectionID == ta.getDocumentCollectionID();
1024        //
1025        // /* spans must be equal -- all spans */
1026        // boolean equalSpans = true;
1027        // List<Span> spans = ta.getSpans();
1028        // if (spanList.size() != spans.size()) {
1029        // equalSpans = false;
1030        // } else {
1031        // for (int i = 0; i < spanList.size(); i++) {
1032        // if (!spanList.get(i).equals(spans.get(i))) {
1033        // equalSpans = false;
1034        // break;
1035        // }
1036        // }
1037        // }
1038        //
1039        // return equalSpans & equalDocumentID & equalDocumentCollectionID;
1040        //
1041        // }
1042    
1043        // public int compareTo(Object textAnnotationToCompare) {
1044        // if (!(textAnnotationToCompare instanceof TextAnnotation)) {
1045        // throw new ClassCastException("A TextAnnotation object expected.");
1046        // } else {
1047        //
1048        // return this.getFirstSpanStartIndex() - ((TextAnnotation) textAnnotationToCompare).getFirstSpanStartIndex();
1049        // }
1050        // }
1051    
1052        // /**
1053        // * must have overlapping span plus matching documentID, docCollectionID, etc.
1054        // * @return
1055        // */
1056        // public boolean strictlyOverlaps(TextAnnotation ta, boolean ignoreClass) {
1057        // /* document IDs must be equal */
1058        // boolean equalDocumentID = this.documentID.equals(ta.getDocumentID());
1059        //
1060        // /* document collection ids must be equal */
1061        // boolean equalDocumentCollectionID = this.documentCollectionID == ta.getDocumentCollectionID();
1062        //
1063        // /* spans must be equal -- all spans */
1064        // boolean overlappingSpans = overlaps(ta);
1065        //        
1066        // /*
1067        // * class mention must be equal - we currently only look at the top level class - this should look at all slots as well in the
1068        // * future
1069        // */
1070        //        
1071        // boolean equalClassMention = false;
1072        // if (ignoreClass) {
1073        // equalClassMention = true;
1074        // } else {
1075        // equalClassMention = this.classMention.equals(ta.getClassMention());
1076        // }
1077        //
1078        // return overlappingSpans & equalClassMention & equalDocumentID & equalDocumentCollectionID;
1079        // }
1080    }
```
